# Comprehensive Evaluation of the Physiological Responses and Cold Tolerance of Annual Shoots from Different Sweet Cherry (*Prunus avium* L.) Cultivars Under Low-Temperature Stress

**DOI:** 10.3390/ijms27167142

**Published:** 2026-08-09

**Authors:** Dilraba Muhtar, Abduxukur Yakup, Wenwen Li, Ablimit Tohti, Yan Zhang, Mansur Nasir

**Affiliations:** 1 College of Horticulture, Xinjiang Agricultural University, Urumqi 830052, China; 18099621925@163.com (D.M.); liwenwen0323@126.com (W.L.); 2 Xinjiang Engineering Technology Research Center for Efficient Cultivation and High-Value Utilization of Forest Fruits, Urumqi 830052, China; 3 Xinjiang Academy of Forestry Sciences, Urumqi 830063, China; komulxj@163.com; 4 National Improved Variety Base of Economic Forest Tree Species, Jiamu Experimental Station, Xinjiang Academy of Forestry Sciences, Jiamu 831100, China; 5 Southern Xinjiang Gobi Facility Fruit Tree Technology Innovation Center, Hotan 848000, China; 6 Aksu Agricultural Science and Technology Innovation Center, Aksu 843000, China; ablimittohti01@163.com (A.T.); rose0907@163.com (Y.Z.)

**Keywords:** sweet cherry, cold tolerance, physiological response, principal component analysis, membership function, LT_50_, recovery growth

## Abstract

Sweet cherry (*Prunus avium* L.) is a high-value horticultural crop; however, winter freezing injury significantly limits its introduction and stable production in cold-temperate regions. In this study, annual shoots of 16 major sweet cherry cultivars were collected during the dormancy phase and subjected to a simulated low-temperature gradient. Eight physiological indicators, namely, relative electrolyte conductivity (REC), malondialdehyde (MDA) content, osmoregulatory substances, and antioxidant enzyme activities, were systematically measured along with the poststress recovery growth percentage (RP). The semilethal temperature (LT_50_) was calculated using a logistic equation, and a multidimensional comprehensive evaluation system for cold tolerance was constructed using the membership function method, principal component analysis (PCA), and cluster analysis. The results indicated that low-temperature stress significantly induced membrane lipid peroxidation and osmotic compensation in the shoots. PCA revealed four principal components—osmoregulation potential, cell membrane damage threshold, cold-protective protein synthesis, and membrane stability—with a cumulative variance contribution rate of 79.47%. Correlation analysis revealed no significant correlation between LT_50_ (reflecting thermodynamic survival capacity) and the recovery growth percentage (reflecting poststress regenerative capacity), suggesting that “passive endurance” and “active recovery” in sweet cherries are two relatively independent physiological processes. The comprehensive evaluation model classified the 16 cultivars into four cold-tolerance tiers: High Tolerance (Russia 8, Reid, Pacific Red, and Brooks); Moderate-High Tolerance (Tieton, Jiahong, Sandra Rose, and Rivedel); Moderate-Low Tolerance (Luyu, Rocket, Tamara, Lapins, Frisco, and Taisho-nishiki); and Sensitive (Summit and Kordia). The four-dimensional evaluation model developed in this study provides a scientific basis for cultivar selection in cold regions and offers a theoretical reference for the trade-off of traits in future molecular breeding for cold resistance in cherries.

## 1. Introduction

Sweet cherry (*Prunus avium* L.) is a member of the *Prunus* genus in the *Rosaceae* family and is a vital deciduous fruit tree in northern temperate regions. Its fruits are highly valued for their vibrant color, unique flavor, and rich content of vitamins and saccharides, significantly contributing to both food nutrition and market economic value [1]. However, with the intensification of global climatic fluctuations, abiotic stressors have become primary factors limiting the geographical distribution and yield stability of sweet cherries [2,3,4]. Among these factors, low-temperature injury has the most profound effect on plant growth and development, frequently leading to shoot dieback during dormancy, yield reduction during anthesis and young fruit stages, and even whole-tree mortality [5,6]. Therefore, elucidating the physiological response mechanisms of sweet cherry to cold stress is highly important for scientific cultivar selection and regionalized cultivation.

The cell membrane system is the primary site of cold perception and injury in plants. Early research by Levitt revealed that acute cellular dehydration induced by freezing is a major factor in membrane damage [6]. Through physicochemical analysis, Steponkus subsequently demonstrated that the cryostability of the plasma membrane during freezing directly determines the survival threshold of cells, as low temperatures alter the physical state of membrane lipids and trigger lipid peroxidation [7,8]. In sweet cherries, the loss of membrane integrity under cold stress leads to intracellular electrolyte leakage, which significantly increases relative electrolyte conductivity (REC). Consequently, the REC has become one of the most direct physiological indicators for evaluating plant cold hardiness [9,10]. Recent studies have further clarified that maintaining membrane homeostasis depends not only on changes in lipid composition but also on rhythmic fluctuations in signaling molecules; for instance, melatonin and its precursors play key roles in signal transduction to regulate stress responses and alleviate environmental stressors [11].

To withstand cold environments, plants have evolved sophisticated metabolic compensatory mechanisms. Primarily, the accumulation of osmotic adjustment substances—such as soluble proteins, proline, and soluble sugars—protects the endomembrane system by enhancing cellular water-holding capacity and stabilizing ice crystals [12,13]. Notably, proline plays a dual role: it functions not only as an osmolyte to maintain water balance but also as an antioxidant and energy carrier, providing the necessary energy for plant growth recovery once the stress subsides [14]. Furthermore, cold stress triggers the excessive accumulation of reactive oxygen species (ROS), which can cause severe oxidative damage if not promptly scavenged [15]. In response, plants activate an enzymatic defense system comprising superoxide dismutase (SOD), peroxidase (POD), and catalase (CAT) to synergistically scavenge free radicals and increase resilience [16]. Recent meta-analyses have indicated that the cold acclimation capacity of woody perennials during dormancy is, in fact, a highly coordinated temporal response involving these antioxidant and osmotic mechanisms [17].

Despite extensive research on cold hardiness in various species, this trait is a complex phenomenon controlled by multiple genes, making it difficult for single physiological indicators to objectively reflect differences in intercultivar resistance. Current evaluation frameworks are shifting from single-index assessments to multidimensional quantitative models. The semilethal temperature (LT_50_) derived from the logistic equation combined with mathematical modeling can effectively overcome the randomness of transient physiological fluctuations [18]. Furthermore, the use of principal component analysis (PCA) and the membership function method to evaluate multiple indicators has proven to be a robust approach for assessing the stress resistance of fruit tree germplasms [19].

In this context, the present study used the annual dormant shoots of 16 major sweet cherry cultivars to systematically characterize their physiological and biochemical dynamics through simulated gradient low-temperature treatments. The objectives were (1) to clarify the synergistic roles of membrane damage, osmotic adjustment, and antioxidant enzyme systems in the response of sweet cherry to cold stress; (2) to construct a multidimensional comprehensive evaluation model using PCA and membership functions; and (3) to precisely identify the cold-hardiness levels across different cultivars. This research aimed to provide a reliable biological foundation for the scientific strategic layout and selection of cold-resilient sweet cherry cultivars in cold regions.

## 2. Results

### 2.1. Classification of Cultivar Cold Tolerance Based on LT_50_ and Membership Function Analysis

As shown in Table 1, the determination coefficients (*R^2^*) of the logistic regression models for the 16 sweet cherry cultivars ranged from 0.941 to 0.995, indicating that the fitting equations were highly reliable. The semilethal temperature (LT_50_) varied notably among the different cultivars. ‘Pacific Red’ had the lowest LT_50_ (−25.7 °C), followed by ‘Russia-8’ (−24.8 °C), while ‘Kordia’ had the highest LT_50_ (−17.4 °C), suggesting that it was the most sensitive to cold stress. On the basis of the LT_50_ values, the cold tolerances of the 16 cultivars were initially ranked in descending order as follows: ‘Pacific Red’ > ‘Russia-8’ > ‘Brooks’ > ‘Reid’ > ‘Frisco’ > ‘Rocket’ > ‘Sandra Rose’ > ‘Jiahong’ > ‘Lapins’ > ‘Tamara’ > ‘Taisho-nishiki’ > ‘Luyu’ > ‘Tieton’ > ‘Rivedel’ > ‘Summit’ > ‘Kordia’.

To gain a more comprehensive understanding of their physiological responses, a membership function analysis based on eight physiological indices was conducted (Table 2). The average membership values ranged from 0.242 to 0.661 and decreased in the order of ‘Pacific Red’ > ‘Russia-8’ > ‘Brooks’ > ‘Reid’ > ‘Tieton’ > ‘Taisho-nishiki’ > ‘Jiahong’ > ‘Rivedel’ > ‘Sandra Rose’ > ‘Frisco’ > ‘Luyu’ > ‘Tamara’ > ‘Rocket’ > ‘Lapins’ > ‘Kordia’ > ‘Summit’. On the basis of these values, and according to the authors’ own classification criteria derived from the mathematical clustering of the dataset, the 16 cultivars were classified into three distinct groups:

1. The cold-resistant group (average membership values ranging from 0.541 to 0.661) included ‘Pacific Red’, ‘Russia-8’, ‘Brooks’, and ‘Reid’.
2. The moderately cold-resistant group (average membership values ranging from 0.421 to 0.489) included ‘Tieton’, ‘Taisho-nishiki’, ‘Jiahong’, ‘Rivedel’, ‘Sandra Rose’, ‘Frisco’, and ‘Luyu’.
3. The cold-sensitive group (average membership values ranging from 0.242 to 0.355): included ‘Tamara’, ‘Rocket’, ‘Lapins’, ‘Kordia’, and ‘Summit’.

‘Tieton’ was selected as the control cultivar because it is currently one of the most widely planted cultivars in the regional cultivation area of northern China (where the experimental material was collected). Its cold tolerance and field performance in these regions are well-documented and widely recognized by local growers, making it a reliable reference standard (CK) for evaluating the cold tolerance of newly introduced or less-studied cultivars.

### 2.2. Effects of Low-Temperature Treatment on Indicators Related to Cell Membrane Damage in Twigs

#### 2.2.1. Relative Electrical Conductivity (REC)

**Figure 1:**
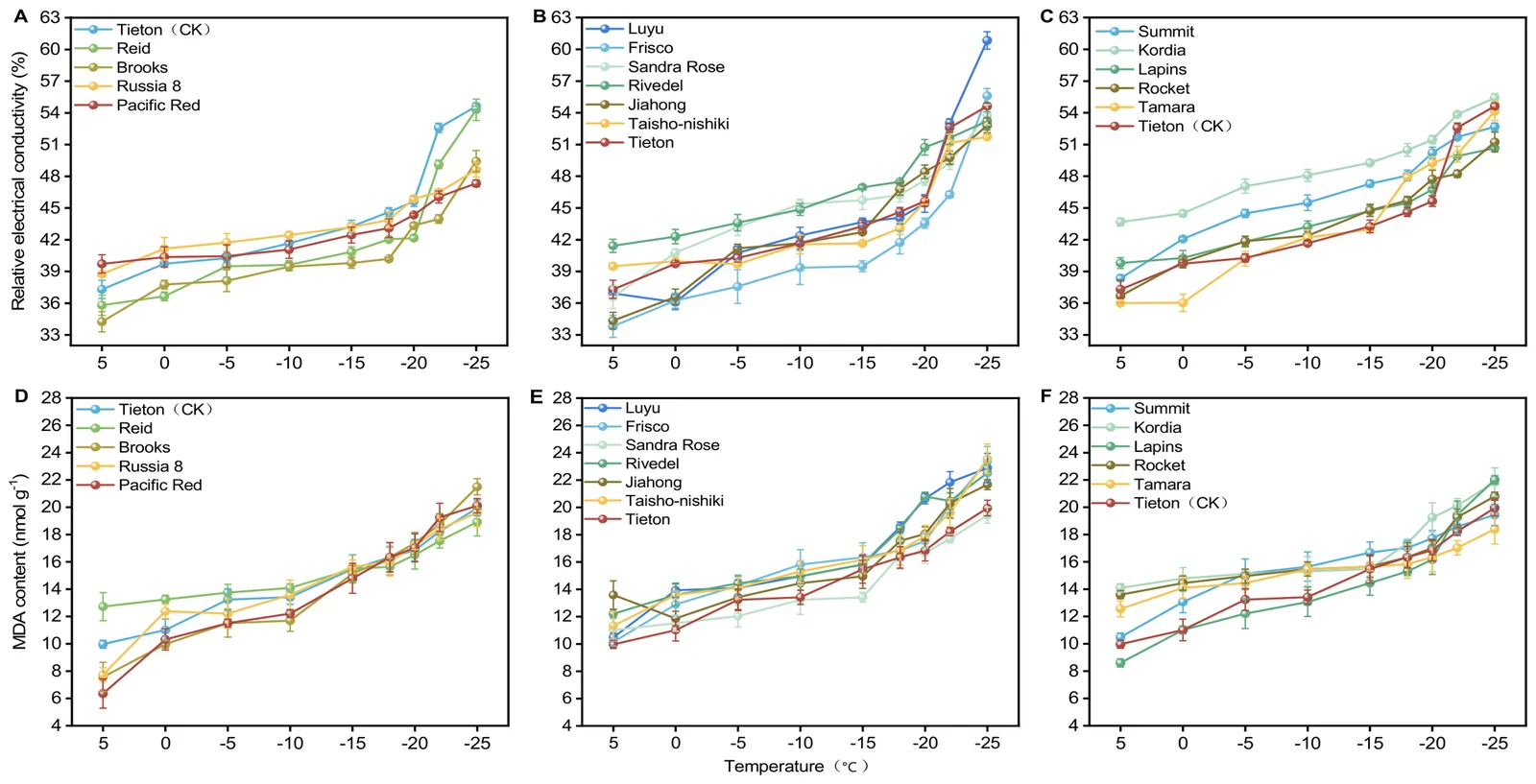
Effects of low-temperature stress on the biological indicators of cell membrane damage in the annual shoots of 16 sweet cherry cultivars. (**A**,**D**) Cold-tolerant group; (**B**,**E**) moderately cold-resistant group; (**C**,**F**) cold-sensitive group. The grouping was based on average membership function values: the cold-tolerant group (0.541–0.661) included ‘Pacific Red’, ‘Russia 8’, ‘Brooks’, and ‘Reid’; the moderately cold-resistant group (0.421–0.489) included ‘Taisho-nishiki’, ‘Jiahong’, ‘Rivedel’, ‘Sandra Rose’, ‘Frisco’, and ‘Luyu’; and the cold-sensitive group (0.242–0.355) included ‘Tamara’, ‘Rocket’, ‘Lapins’, ‘Kordia’, and ‘Summit’. ‘Tieton’ served as the comparative control (CK) for each group. The line colors represent the magnitude of the average membership values, transitioning from cool colors (blue/green, representing lower membership values) to warm colors (yellow/red, representing higher membership values). Error bars represent the standard error (SE) of the mean from three biological replicates (*n* = 3) The same abbreviations and representation conventions apply below.

REC is a critical indicator of cell membrane permeability and the extent of cell membrane damage under freezing stress. The dynamic changes in REC for the annual shoots of 16 sweet cherry cultivars under different low-temperature treatments (from 5 to −25 °C) are illustrated in Figure 1A–C. As the treatment temperature decreased, the REC of all the tested cultivars exhibited a consistent and progressive upward trend. From 5 to −15 °C, the REC of most cultivars increased gradually and remained relatively low (below 45%), suggesting that the cell membrane structure remained relatively intact within this temperature range. However, when the temperature fell below −15 °C, the REC values increased sharply, indicating that the freezing temperatures exceeded the tolerance threshold of the cell membranes, resulting in substantial leakage of intracellular electrolytes. Slightly different response patterns were observed among the three groups.

In the cold-tolerant group (Figure 1A), the cultivars maintained overall lower REC levels throughout the cooling process. At −25 °C, ‘Pacific Red’ and ‘Russia-8’ showed the lowest REC values (47.2% and 49.3%, respectively), demonstrating superior cell membrane stability compared with that of the control cultivar ‘Tieton’ (54.5%).

In the moderately cold-resistant group (Figure 1B), the curves of the cultivars clustered closely with those of the control ‘Tieton’. At −25 °C, most cultivars reached REC values between 51.0% and 54.0%, whereas ‘Luyu’ had a slightly higher REC, approaching 58%.

In the cold-sensitive group (Figure 1C), the REC of the cultivars increased rapidly at subzero temperatures. Notably, ‘Summit’ was extremely sensitive to negative temperatures; its REC value started at a high flat phase at 5 to −15 °C and soared to the highest level among all the cultivars (61.2%) at −25 °C, followed by ‘Kordia’ (53.6%).

These findings indicate that cold-tolerant cultivars possess a stronger ability to maintain cell membrane integrity under freezing stress.

#### 2.2.2. Malondialdehyde (MDA) Content

Malondialdehyde (MDA) is the end product of membrane lipid peroxidation, and its accumulation reflects the level of oxidative stress damage to cell membranes [20,21]. The accumulation of MDA in the annual shoots of the 16 sweet cherry cultivars under low-temperature stress is shown in Figure 1D–F. Under normal or mild chilling conditions (5 °C), the initial MDA content of all the cultivars was relatively low, ranging from 6.31 to 13.56 nmol g^−1^. As the stress temperature steadily decreased, the MDA levels in all the cultivars gradually increased, reflecting the progressive lipid peroxidation of cell membranes under low-temperature stress. The accumulation kinetics of MDA varied distinctly among the different groups.

In the cold-tolerant group (Figure 1D), the rate of MDA accumulation was relatively slow. The cold-tolerant cultivar ‘Pacific Red’ maintained the lowest levels of MDA throughout the experiment. Even under the extreme temperature of −25 °C, the MDA content reached only 20.12 nmol g^−1^, which was lower than that of the control ‘Tieton’ (21.05 nmol g^−1^), indicating a minor degree of membrane damage in this cultivar.

In the moderately cold-resistant group (Figure 1E), the cultivars showed intermediate levels of MDA accumulation. With decreasing temperature, the MDA content increased steadily. At −25 °C, the MDA content of most cultivars in this group ranged from 19.50 to 23.50 nmol g^−1^, with ‘Luyu’ and ‘Frisco’ showing slightly higher levels than the other cultivars did.

In the cold-sensitive group (Figure 1F), the MDA content of the sensitive cultivar increased rapidly under freezing stress. At −25 °C, the MDA contents of ‘Summit’ and ‘Kordia’ increased to peak values of 22.04 nmol g^−1^ and 20.83 nmol g^−1^, respectively.

In summary, the results show that MDA accumulation under low-temperature stress was less pronounced in cold-tolerant cultivars compared to cold-sensitive ones, indicating differences in membrane lipid stability among the cultivars during cold exposure.

### 2.3. Changes in the Levels of Osmoregulatory Substances Under Cold Stress

#### 2.3.1. Soluble Protein

**Figure 2:**
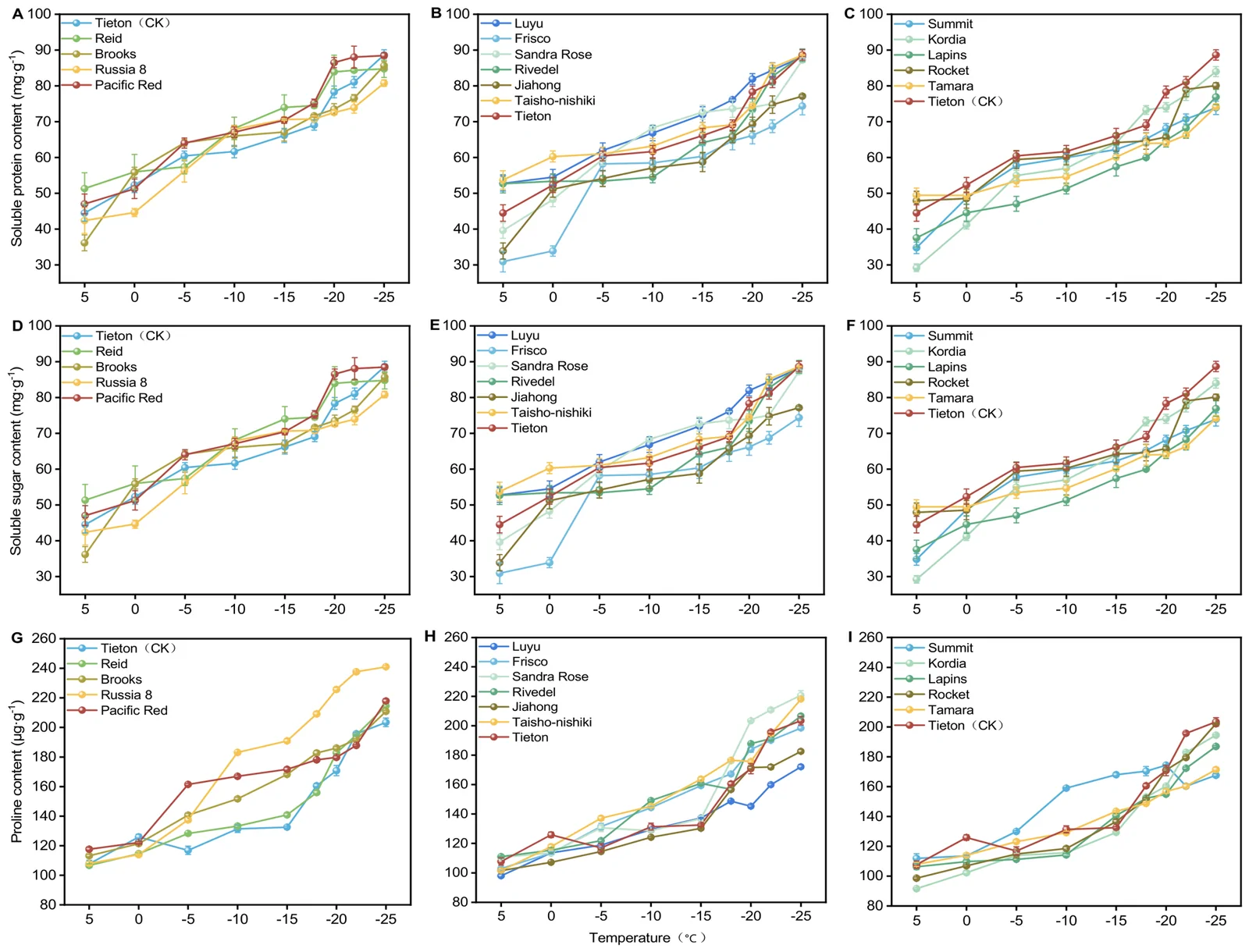
Effects of low-temperature stress on the content of osmoregulatory substances in the annual shoots of 16 sweet cherry cultivars. (**A**–**C**) Soluble protein content; (**D**–**F**) soluble sugar content; (**G**–**I**) proline content. The groupings are as follows: (**A**,**D**,**G**) cold-tolerant group; (**B**,**E**,**H**) moderately cold-resistant group; (**C**,**F**,**I**) cold-sensitive group. The same abbreviations and representation conventions apply below.

Soluble proteins play dual roles as osmotic regulators and protective proteins for cellular structures during cold acclimation. The changes in the soluble protein content of the 16 cultivars are shown in Figure 2A–C. With decreasing treatment temperature from 5 to −25 °C, the soluble protein content of all the cultivars exhibited a continuous, steady upward trend. Under control conditions (5 °C), the initial soluble protein content across all the cultivars ranged between 30.29 and 52.88 mg g^−1^. As the temperature decreased, the proteins accumulated steadily.

In the cold-tolerant group (Figure 2A), the cultivars accumulated soluble proteins rapidly. At −25 °C, ‘Russia-8’ and ‘Pacific Red’ had high protein concentrations of approximately 88.24 mg g^−1^ and 87.85 mg g^−1^, respectively, which were higher than or comparable to those of the control cultivar ‘Tieton’ (85.22 mg g^−1^).

In the moderately cold-resistant group (Figure 2B), most of the cultivars (such as ‘Frisco’ and ‘Taisho-nishiki’) showed a parallel increase. ‘Frisco’ started with a lower baseline (30.91 mg g^−1^) but increased sharply at subzero temperatures, reaching 74.65 mg g^−1^ at −25 °C.

In the cold-sensitive group (Figure 2C), the cultivars generally maintained lower levels at the early stages of cold stress. Although they accumulated proteins at later stages, their peak values at −25 °C were relatively low, except for ‘Tamara’, which reached 89.04 mg g^−1^.

#### 2.3.2. Soluble Sugar

Soluble sugars serve as both major energy sources and effective cryoprotectants that hinder ice crystal formation within plant tissues. The changes in soluble sugar content under cold stress are illustrated in Figure 2D–F. Like the trend of soluble proteins, the soluble sugar content in all 16 cultivars increased progressively as the temperature decreased. At 5 °C, the initial sugar levels were tightly clustered between 30.15 and 51.48 mg g^−1^.

In the cold-tolerant group (Figure 2D), the rate of sugar accumulation in ‘Russia-8’ and ‘Pacific Red’ was high, increasing sharply past −15 °C and reaching peak values of 88.02 mg g^−1^ and 87.94 mg g^−1^, respectively, at −25 °C.

In the moderately cold-resistant group (Figure 2E), the sugar content profiles of the cultivars tracked closely with each other. Cultivars in this group converged within a range of 73.50 to 88.80 mg g^−1^ at −25 °C, with ‘Tieton’ having the highest value (88.13 mg g^−1^).

In the cold-sensitive group (Figure 2F), a lagging accumulation pattern was observed. For instance, ‘Summit’ maintained lower sugar concentrations at 5 to −15 °C, only markedly increasing at extreme temperatures (−20 to −25 °C) and ending at 74.22 mg g^−1^.

#### 2.3.3. Proline

Proline is a highly efficient osmolyte that also functions as a reactive oxygen species (ROS) scavenger under environmental anomalies. The response of the proline content to decreasing temperature is highlighted in Figure 2G–I. Unlike the gradual increase in proteins and sugars, proline accumulation exhibited a distinct “slow-then-rapid” pattern, with an abrupt increase occurring below −15 °C. At 5 °C, the baseline proline levels of all the cultivars were low and uniform (90.28 to 118.42 μg g^−1^).

In the cold-tolerant group (Figure 2G), ‘Russia-8’ displayed an extraordinary capacity for proline synthesis, with its content rising dramatically from 118.41 μg g^−1^ (at 0 °C) to 241.15 μg g^−1^ (at −25 °C). This was the highest value recorded among all the cultivars, followed closely by ‘Pacific Red’ (218.45 μg g^−1^) and ‘Tieton’ (209.60 μg g^−1^).

In the moderately cold-resistant group (Figure 2H), the cultivars remained clustered until −15 °C, after which they diverged. At −25 °C, ‘Sandra Rose’ and ‘Frisco’ exhibited high levels (~220.12 μg g^−1^), whereas ‘Luyu’ was significantly lower (170.85 μg g^−1^).

In the cold-sensitive group (Figure 2I), under freezing conditions (−25 °C), these cultivars exhibited slow proline accumulation. Notably, ‘Summit’ reached only 167.35 μg g^−1^, which was significantly lower than the values observed in the cold-tolerant group, highlighting a deficient osmotic adjustment response under extreme cold stress.

### 2.4. Effects of Low-Temperature Stress on Antioxidant Enzyme Activities

#### 2.4.1. Superoxide Dismutase (SOD)

**Figure 3:**
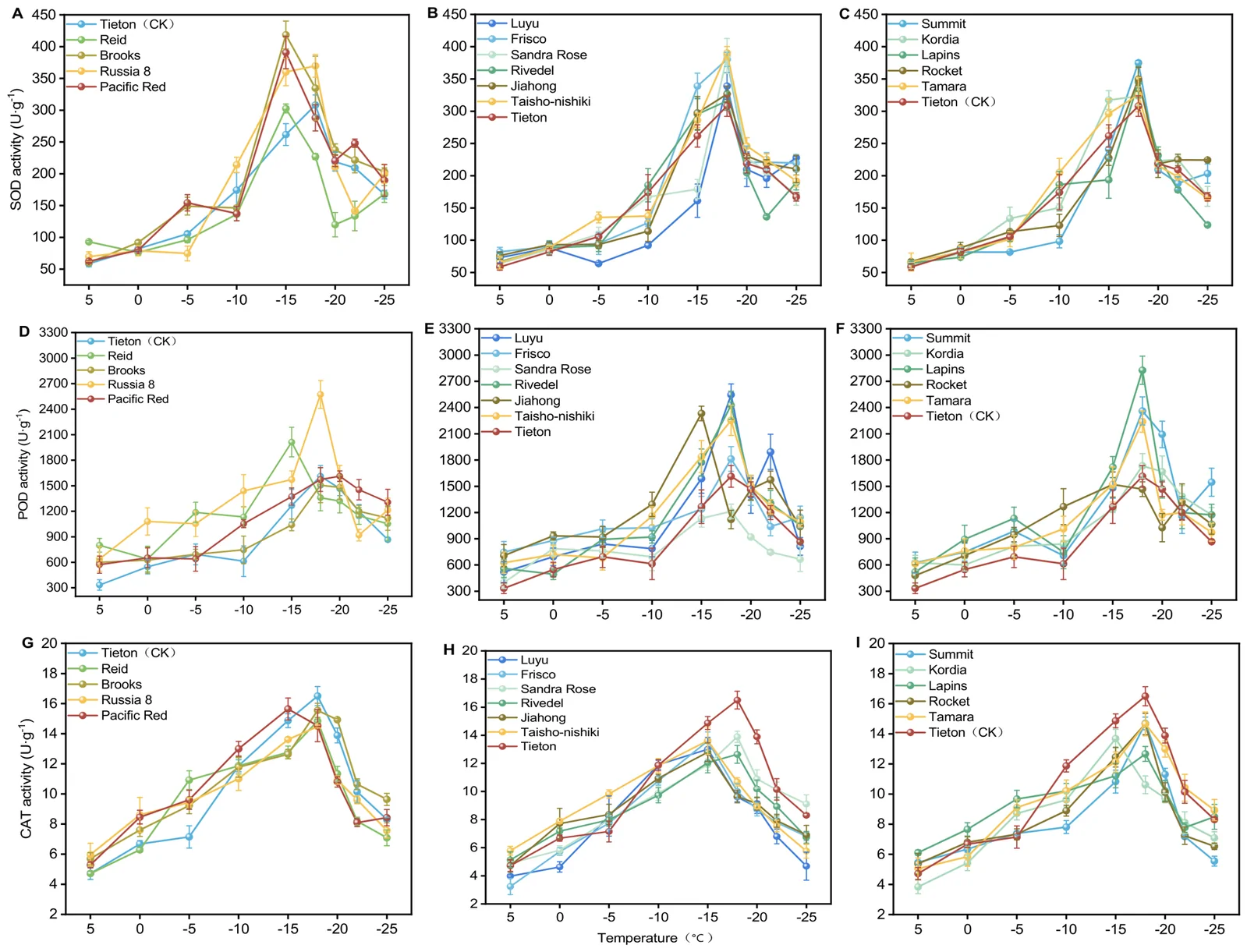
Effects of low-temperature stress on the activities of antioxidant enzymes in the annual shoots of 16 sweet cherry cultivars. (**A**–**C**) Superoxide dismutase (SOD) activity; (**D**–**F**) peroxidase (POD) activity; (**G**–**I**) catalase (CAT) activity. The groupings are arranged as follows: (**A**,**D**,**G**) cold-tolerant group; (**B**,**E**,**H**) moderately cold-resistant group; (**C**,**F**,**I**) cold-sensitive group. Effects of low-temperature stress on antioxidant enzyme activity.

SOD serves as the first line of defense against oxidative stress by catalyzing the dismutation of superoxide radicals (O^2−^) into hydrogen peroxide (H_2_O_2_) and oxygen (O_2_). The dynamic changes in SOD activity are shown in Figure 3A–C. With decreasing temperature from 5 to −25 °C, the SOD activity of all the cultivars exhibited a distinct unimodal (single-peak) curve. At 5 °C, the initial SOD activity was relatively low, ranging from 50.12 to 89.45 U g^−1^. As the stress intensified, the SOD activity increased progressively, peaking at −15 °C or −18 °C.

In the cold-tolerant group (Figure 3A), the cultivars responded rapidly to low temperatures. ‘Russia 8’ and ‘Pacific Red’ reached their peak activities at −15 °C (415.20 and 388.50 U g^−1^, respectively), which were greater than those of the control ‘Tieton’ (302.30 U g^−1^), reflecting a highly sensitive and robust primary defense response.

In the moderately cold-resistant group (Figure 3B), most of the cultivars peaked at −18 °C. ‘Frisco’ and ‘Sandra Rose’ exhibited prominent peak activities of 395.40 and 385.10 U g^−1^, respectively.

In the cold-sensitive group (Figure 3C), the SOD activity also peaked at −18 °C. Notably, ‘Summit’ reached its peak activity of 368.50 U g^−1^ at −18 °C but decreased sharply to 123.40 U g^−1^ at −25 °C, indicating that extreme freezing temperatures severely damaged the enzyme structure or inhibited its synthesis.

#### 2.4.2. Peroxidase (POD)

POD is responsible for scavenging H_2_O_2_ in the extracellular space and vacuoles. The changes in POD activity under cold stress are illustrated in Figure 3D–F. Like the activity of SOD, the POD activity in all the tested cultivars followed a unimodal trend, increasing first and then decreasing as the temperatures decreased.

In the cold-tolerant group (Figure 3D), ‘Russia 8’ demonstrated a markedly high activity response, with POD activity increasing rapidly and peaking at −18 °C, with a value of 2580.40 U g^−1^. ‘Pacific Red’ and ‘Tieton’ also maintained high values at their peaks (~1500 to 1600 U g^−1^).

The moderately cold-resistant group (Figure 3E), the cultivars peaked uniformly at −18 °C. ‘Frisco’ and ‘Taisho-nishiki’ showed strong enzymatic activity, peaking at 2405.00 and 2280.00 U g^−1^, respectively, before decreasing under the colder treatments.

In the cold-sensitive group (Figure 3F), ‘Summit’ sharply increased at −18 °C, reaching a peak value of 2820.00 U g^−1^, followed by a precipitous decrease to 880.60 U g^−1^ at −25 °C. The rapid decrease in sensitive cultivars at temperatures below −18 °C suggests that severe cold stress exceeded their regulatory capacity, leading to a collapse of the POD-mediated recycling pathway.

#### 2.4.3. Catalase (CAT)

CAT mainly decomposes high concentrations of H_2_O_2_ in peroxisomes. The response of CAT activity to low-temperature treatments is presented in Figure 3G–I. Consistent with the activity of SOD and POD, the activity of CAT also showed a typical unimodal response, with its activity peaking between −15 °C and −18 °C.

In the cold-tolerant group (Figure 3G), antiseptic activity increased steadily during the initial cooling phase. ‘Pacific Red’ and ‘Reid’ peaked at −15 °C (15.54 and 14.85 U g^−1^, respectively) and remained relatively high at −25 °C compared with those in the other groups.

In the moderately cold-resistant group (Figure 3H), most of the cultivars reached maximum CAT activity at −18 °C. ‘Tieton’ achieved the highest peak activity in this group (16.20 U g^−1^), while ‘Luyu’ peaked at a lower value of 12.05 U g^−1^.

In the cold-sensitive group (Figure 3I), the activity of the cultivars peaked at −18 °C, with ‘Tamara’ having the highest CAT activity (16.32 U g^−1^). However, the activity in all the sensitive cultivars decreased rapidly at lower temperatures, decreasing to 5.50–8.80 U g^−1^ at −25 °C.

Overall, the coordinated activity of SOD, POD, and CAT during the cooling process highlights the active defense of sweet cherry twigs. Cold-tolerant cultivars maintained higher antioxidant activities at extremely low temperatures, offering better protection against peroxidation.

### 2.5. Effects of Low-Temperature Stress on the Relative Recovery Growth Percentage of Dormant Annual Shoots

#### 2.5.1. Recovery Growth Percentage

The relative recovery growth percentage of annual shoots during the dormant period is a direct phenotypic indicator reflecting tissue viability and the extent of damage following stress. As shown in Table 3, as the treatment temperature decreased from 5 to −25 °C, the recovery growth percentage of the 16 sweet cherry cultivars progressively decreased, indicating significant variation in cold tolerance among the cultivars. Within the temperature range of 5 to −10 °C, the shoots of all cultivars displayed robust survival capability, with the recovery growth percentage of most of the cultivars remaining above 80.0%. Notably, ‘Summit’, ‘Lapins’, and ‘Taisho-nishiki’ maintained a recovery growth percentage of 100.0% at 0 to −5 °C. A distinct inflection point in shoot viability occurred when the temperature decreased to the range of −15 to −20 °C. At −20 °C, the recovery growth percentage ranged from 33.8% (‘Taisho-nishiki’) to 63.6% (‘Luyu’). When the temperature decreased to the lowest value of −25 °C, half of the cultivars completely lost their ability to recover, indicating that this temperature exceeded the cellular tolerance threshold of these cultivars. Conversely, the remaining eight cultivars still retained a degree of viability. Among these, ‘Brooks’ and ‘Pacific Red’ were the most prominent, maintaining a recovery growth percentage of 20.0%, followed by ‘Russia 8’ (18.8%) and ‘Tieton’ (15.9%). To comprehensively evaluate the cold hardiness of these sweet cherry cultivars under winter freezing conditions, the average recovery growth percentage in the critical lethal temperature range (−15 to −25 °C) was calculated. On the basis of the average recovery growth percentage, the cold hardiness of the annual shoots of the 16 sweet cherry cultivars, in descending order, was ‘Luyu’ > ‘Tieton’ > ‘Rocket’ > ‘Reid’ > ‘Rivedel’ > ‘Brooks’ > ‘Pacific Red’ > ‘Sandra Rose’ > ‘Russia 8’ > ‘Summit’ > ‘Lapins’ > ‘Jiahong’ > ‘Tamara’ > ‘Kordia’ > ‘Frisco’ > ‘Taisho-nishiki’. These results demonstrate that the poststress regenerative capacity of dormant annual shoots varies significantly among sweet cherry cultivars, thereby providing a reliable phenotypic basis for screening cold-resistant germplasms in temperate fruit crop production.

#### 2.5.2. Effects of Freezing Stress on the Bud Burst and Leafing Phenotypes of Sweet Cherry Cuttings

**Figure 4:**
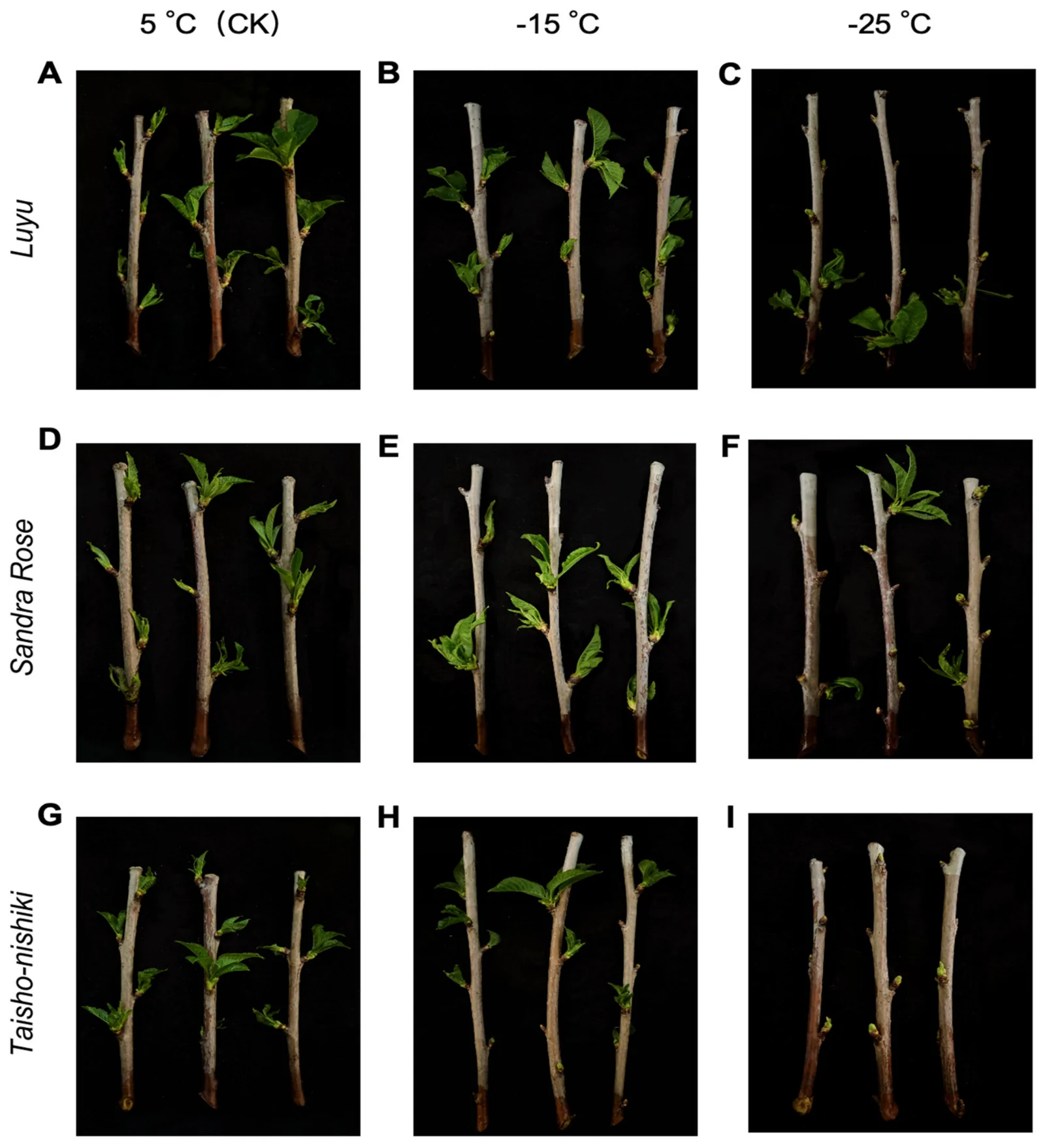
Phenotypic characteristics of bud burst and leaf development in three representative sweet cherry cultivars under different low-temperature treatments. The three cultivars were selected from a screening of 16 cultivars on the basis of their recovery growth percentage, representing distinct cold tolerance levels: Luyu (ranked first, highly cold resistant), Sandra Rose (ranked intermediate, moderately tolerant), and Taisho-nishiki (ranked last, highly cold sensitive). Columns from left to right represent the control (5 °C, CK), −15 °C, and −25 °C treatments, respectively. Rows from top to bottom represent the Luyu (**A**–**C**), Sandra Rose (**D**–**F**), and Taisho-nishiki (**G**–**I**) cultivars.

To visually evaluate the cold tolerance of the sweet cherry cultivars, three representative cultivars—Luyu (cold-resistant, ranked 1st), Sandra Rose (intermediate, ranked middle), and Taisho-nishiki (cold-sensitive, ranked last)—were selected on the basis of their recovery growth percentage after simulated freezing stress (Figure 4).

Under control conditions (5 °C), cuttings of all three cultivars exhibited normal, active bud bursts and healthy green leaf expansion with no signs of damage (Figure 4A,D,G).

When the plants were exposed to −15 °C, distinct cultivar-specific phenotypic variations emerged. The growth and robust leaf expansion of the cold-resistant cultivar Luyu were similar to those of the control group (Figure 4B). The intermediate cultivar Sandra Rose also achieved successful bud burst and leafing, although the leaves were slightly smaller than those of the control (Figure 4E). In contrast, the cold-sensitive Taisho-nishiki cultivar exhibited a visible delay in bud burst, resulting in sparse and small leaves (Figure 4H).

Under severe freezing stress of −25 °C, the differences in cold tolerance became even more pronounced. Luyu demonstrated exceptional freezing tolerance, as evidenced by successful bud burst and visible green leaf elongation even after −25 °C treatment (Figure 4C). Conversely, the development of leaves in Sandra Rose was severely restricted, with only a few stunted leaves emerging (Figure 4F). Crucially, the sensitive cultivar Taisho-nishiki suffered lethal damage at −25 °C, resulting in complete inhibition of bud burst, shoot desiccation, and total failure to develop leaves (Figure 4I).

These phenotypic observations are highly consistent with the recovery growth percentage rankings of the 16 tested cultivars, visually confirming the cold-tolerance gradient: Luyu > Sandra Rose > Taisho-nishiki.

### 2.6. Correlation Analysis Among Physiological Indicators of Cold Hardiness

**Figure 5:**
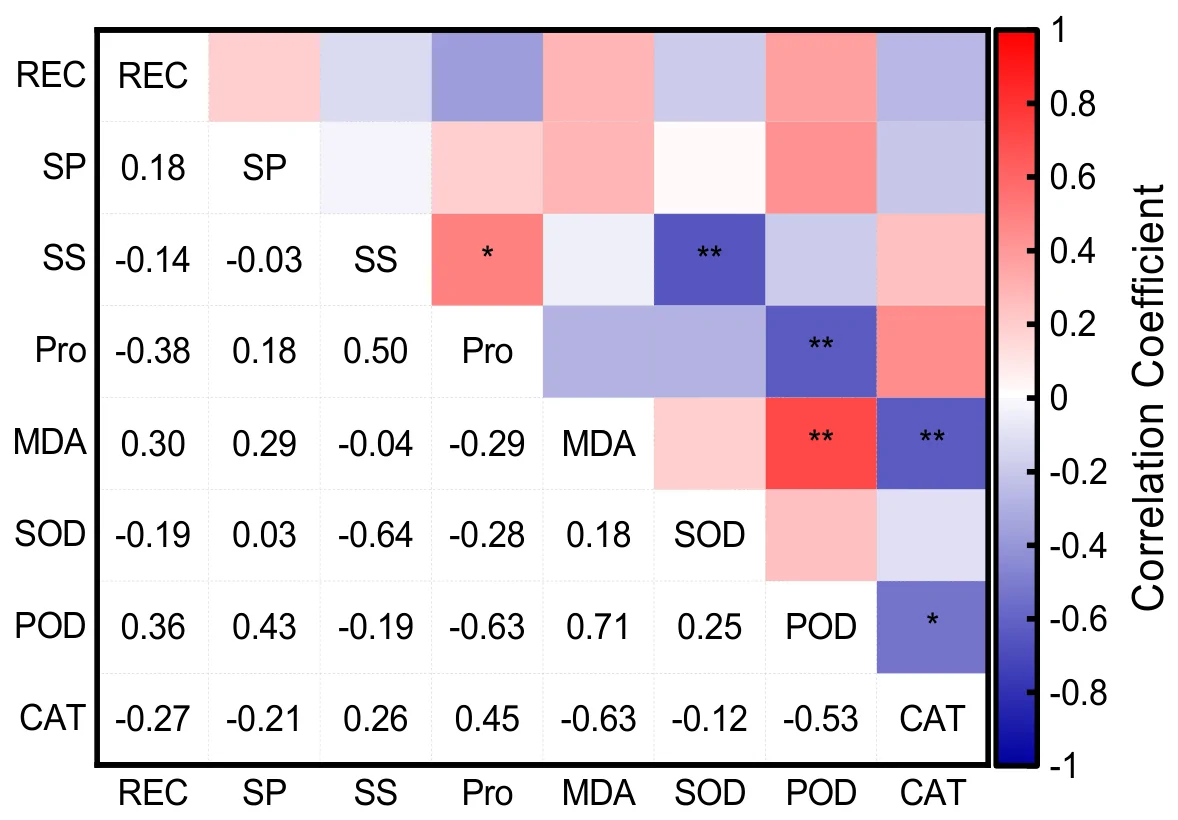
Pearson correlation analysis of the physiological indicators associated with cold tolerance in the annual shoots of succulent sweet cherry. Abbreviations: REC, relative electrical conductivity; SP, soluble protein; SS, soluble sugar; Pro, proline; MDA, malondialdehyde; SOD, superoxide dismutase; POD, peroxidase; CAT, catalase. Asterisks * and ** indicate significant (*p* < 0.05) and highly significant (*p* < 0.01) correlations, respectively.

To determine the intrinsic relationships and synergistic regulatory mechanisms among different physiological indicators of dormant annual shoots of sweet cherry under low-temperature stress, a Pearson correlation analysis was performed (Figure 5). The results demonstrated that the soluble sugar content was significantly and positively correlated with the proline content (*r* = 0.50, *p* < 0.05), indicating that these two core osmoregulants accumulated synergistically under low temperatures to jointly maintain cell osmotic pressure and membrane stability. In terms of membrane lipid peroxidation and the antioxidant system, the malondialdehyde (MDA) content exhibited a highly significant positive correlation with POD activity (*r* = 0.71, *p* < 0.01) and a highly significant negative correlation with CAT activity (*r* = −0.63, *p* < 0.01). These findings suggest that severe membrane damage (indicated by high MDA levels) significantly induced and stimulated POD activity to scavenge reactive oxygen species (ROS), whereas the concurrent low-temperature stress or excessive ROS accumulation may have inhibited CAT activity. Furthermore, a significant negative correlation was detected between POD activity and CAT activity (*r* = −0.53, *p* < 0.05), reflecting a functional compensation or substrate competition relationship between these two H_2_O_2-_scavenging enzymes. Additionally, the proline content was strongly significantly and negatively correlated with POD activity (*r* = −0.63, *p* < 0.01), and the soluble sugar content was strongly significantly and negatively correlated with SOD activity (*r* = −0.64, *p* < 0.01), which suggests the presence of negative feedback regulation between the osmoregulatory and enzymatic antioxidant systems in terms of energy allocation or signal transduction. Relative electrical conductivity (REC) and soluble protein content were not significantly correlated with the other indicators (*p* > 0.05), suggesting that they represent relatively independent physiological pathways involved in the response to cold injury.

### 2.7. Principal Component Analysis (PCA) of Cold Tolerance-Related Indicators

To integrate the multidimensional physiological responses of sweet cherry annual shoots under low-temperature stress and reduce data redundancy, a principal component analysis (PCA) was performed on the eight primary physiological indicators (Table 4). According to the Kaiser criterion, principal components (PCs) with eigenvalues ≥1 are typically selected. The first three principal components (PC1–PC3) exhibited eigenvalues greater than 1, yielding a cumulative variance contribution rate of 67.700%. To retain more comprehensive information (targeting a cumulative variance close to 80%), the fourth principal component (PC4, eigenvalue = 0.941) was included in the analysis, which increased the cumulative contribution rate to 79.466% (Table 4). These results demonstrate that these four PCs effectively and comprehensively represent the majority of the physiological characteristics of cold hardiness in dormant sweet cherry shoots.

The first principal component (PC1) explained the greatest proportion of the total variance (35.649%, eigenvalue = 2.852). In PC1, proline (0.473), CAT (0.417), and soluble sugar (0.336) exhibited high positive loadings, whereas POD (−0.430) and MDA (−0.373) showed strong negative loadings. These findings indicate that PC1 primarily reflects the osmoregulatory and reactive oxygen species (ROS) scavenging capacity of the plant. A higher positive PC1 value indicates a stronger capacity for osmotic maintenance and enzymatic defense under cold stress. The second principal component (PC2) explained 18.789% of the variance (eigenvalue = 1.503), characterized by dominant positive loadings for soluble sugars (0.533), MDA (0.358), and REC (0.349), alongside a strong negative loading for SOD (−0.597). Thus, PC2 reflects mainly the interaction between cell membrane permeability damage and carbohydrate signaling. The third (PC3) and fourth (PC4) principal components explained 13.262% and 11.765% of the total variance, respectively. In PC3, soluble protein exhibited the highest positive loading (0.777), highlighting the critical role of cold-induced proteins in protecting cells from ice crystal injury. PC4 was dominated by REC (0.655), directly reflecting the permeability threshold of cell membranes under extremely low temperatures. In summary, PCA successfully simplified the eight complex physiological variables into four independent comprehensive indicators representing distinct physiological mechanisms (osmotic adjustment, ROS homeostasis, membrane damage, and cryoprotective protein synthesis), establishing an objective weight basis for the subsequent quantitative evaluation of cold hardiness.

Using the variance contribution rate of each principal component as a weighting coefficient, a comprehensive model for evaluating cold hardiness was constructed as follows:

(1) $$F=\frac{35.649\times F_{1}+18.789\times F_{2}+13.262\times F_{3}+11.765\times F_{4}}{79.466}$$

On the basis of this formula, the comprehensive *F*-scores and rankings for the 16 sweet cherry cultivars were calculated (Table 5). The results revealed that Russia 8 achieved the highest score (2.205), indicating the strongest cold tolerance, followed by Reid (0.911) and Sandra Rose (0.680). Conversely, Summit (−0.971) and Rocket (−1.061) received the lowest scores, indicating the weakest cold tolerance.

**Figure 6:**
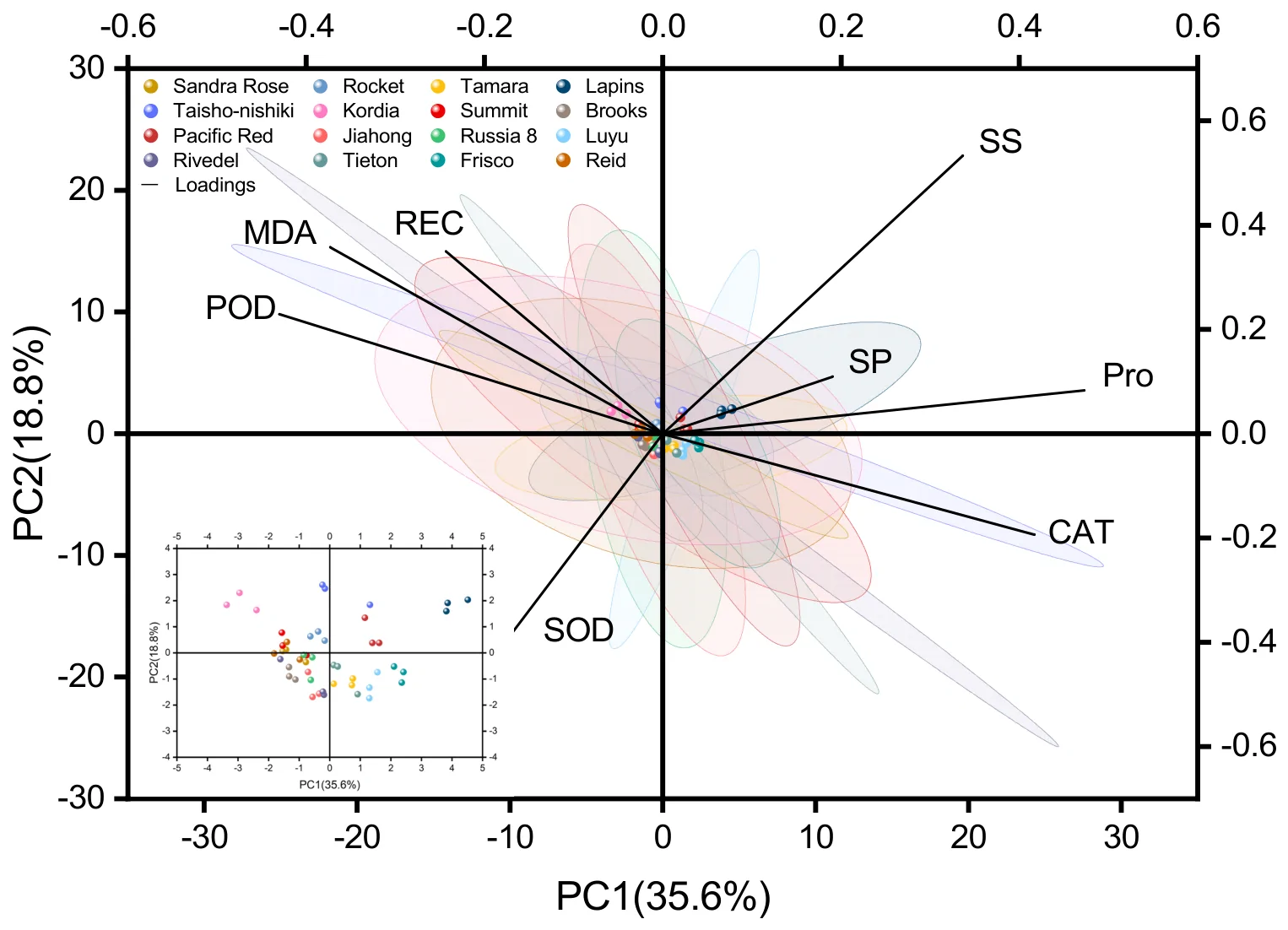
Principal component analysis (PCA) biplot demonstrating the relationships among physiological parameters and the distribution of 16 sweet cherry cultivars under low-temperature stress. The vectors represent the loading of physiological indicators, and the scatter plots represent the scores of individual cultivars.The colored ellipses represent the distribution areas (confidence intervals) of the corresponding 16 sweet cherry cultivars as indicated in the legend.

The relationships among the physiological indicators and the performance of the cultivars were visually evaluated using a PCA biplot (Figure 6). The loading vectors revealed that Pro, CAT, SS, and SP are positioned on the right side of the biplot and are positively correlated with PC1. Conversely, MDA, REC, and POD, which reflect the severity of cell damage, are clustered on the left side and negatively correlated with PC1. In terms of cultivar distribution, Russia 8 is located prominently along the positive axis of PC1, indicating a robust accumulation of osmoregulants and elevated antioxidant enzyme activities, which subsequently minimized its cellular damage under low-temperature stress. In contrast, cultivars such as Rocket and Summit are distributed on the left side (negative along PC1), suggesting a greater susceptibility to cold stress characterized by severe cell membrane damage. Furthermore, the spatial distance between the scatter points of the cultivars reflects the similarity in their physiological profiles; the close proximity between Pacific Red and Frisco indicates that they share highly similar physiological response and adaptation mechanisms under winter freezing conditions.

### 2.8. Comprehensive Evaluation of Cold Tolerance in Sweet Cherry Cultivars Based on the CRITIC Objective Weighting Method

Due to the multipathway and complex biological nature of plant responses to low-temperature stress, relying on a single physiological indicator often introduces evaluation bias. To avoid potential implicit weighting caused by information overlapping among physiological and biochemical indices, the criteria importance through intercriteria correlation (CRITIC) method was used for objective weight allocation. This method determines the information content of each indicator through the product of its contrast intensity (standard deviation) and conflict (independence among indicators, measured as 1 − *r_ij_*).

On the basis of the standard deviations and correlations of the normalized parameters—normalized LT_50_(*N*(LT_50_)), normalized membership degree (*N*(MD)), normalized relative recovery growth percentage (*N*(RP)), and normalized PCA score (*N*(PCA))—the objective weights for the four core evaluation indices were determined to be 21.66%, 21.94%, 32.11%, and 24.29%, respectively. Notably, because the postdamage recovery capacity (*N*(RP)) was statistically decoupled from the survival limit indicators (e.g., LT_50_ and MD), it exhibited the greatest independence (lowest information overlap) and was thus assigned the highest weighting (32.11%) by the CRITIC algorithm. The comprehensive evaluation score (*F*-value) for each cultivar was calculated using the following formula:

(2) $$F=0.2166\times N({LT}_{50})+0.2194\times N(MD)+0.3211\times N(RR)+0.2429\times N(PCA)$$

Based on the recalculated comprehensive evaluation scores (*F*-scores; Table 6), the 16 sweet cherry cultivars were categorized into four cold-tolerance tiers (Table 7):

Tier I—Highly Cold-Tolerant (*F* ≥ 0.65): This tier includes Russia 8, Reid, Pacific Red, and Brooks. Russia 8 ranked first, with an *F*-score of 0.8629, demonstrating outstanding balanced defense, with high scores across all dimensions.

Tier II—Moderately to Highly Cold-Tolerant (0.50 ≤ *F* < 0.65): This tier is composed of Tieton, Sandra Rose, Luyu, and Rivedel. Owing to the high weight of *N*(RP), Luyu (which exhibited a maximum *N*(RP) of 1.0000) was successfully classified into this tier (ranking 7th, *F* = 0.5393), demonstrating that its excellent recovery capacity compensatively bolstered its comprehensive hardiness.

Tier III—Moderately to Weakly Cold-Tolerant (0.30 ≤ *F* < 0.50): This tier comprises Jiahong, Rocket, Tamara, Lapins, Frisco, and Taisho-nishiki. Although Jiahong displayed fair biochemical resilience (*N*(MD) = 0.5537), its moderate recovery capacity (*N*(RP) = 0.5717) restricted its overall ranking.

Tier IV—Cold-Sensitive (*F* < 0.30): This tier includes Summit and Kordia. In particular, Kordia was identified as the most cold-sensitive cultivar (*F* = 0.2024), characterized by poor membrane cryoprotection (*N*(LT_50_) = 0.0000) and a delayed osmoregulatory response (*N*(MD) = 0.0644).

Therefore, when sweet cherry cultivars are selected for regions with high freezing risk, Tier I cultivars are highly recommended. Moreover, Tier II cultivars, characterized by rapid postfrost recovery (e.g., ‘Luyu’), represent key germplasms for local spring frost mitigation.

### 2.9. Correlation Analysis of Cold Tolerance Evaluation Indices

**Figure 7:**
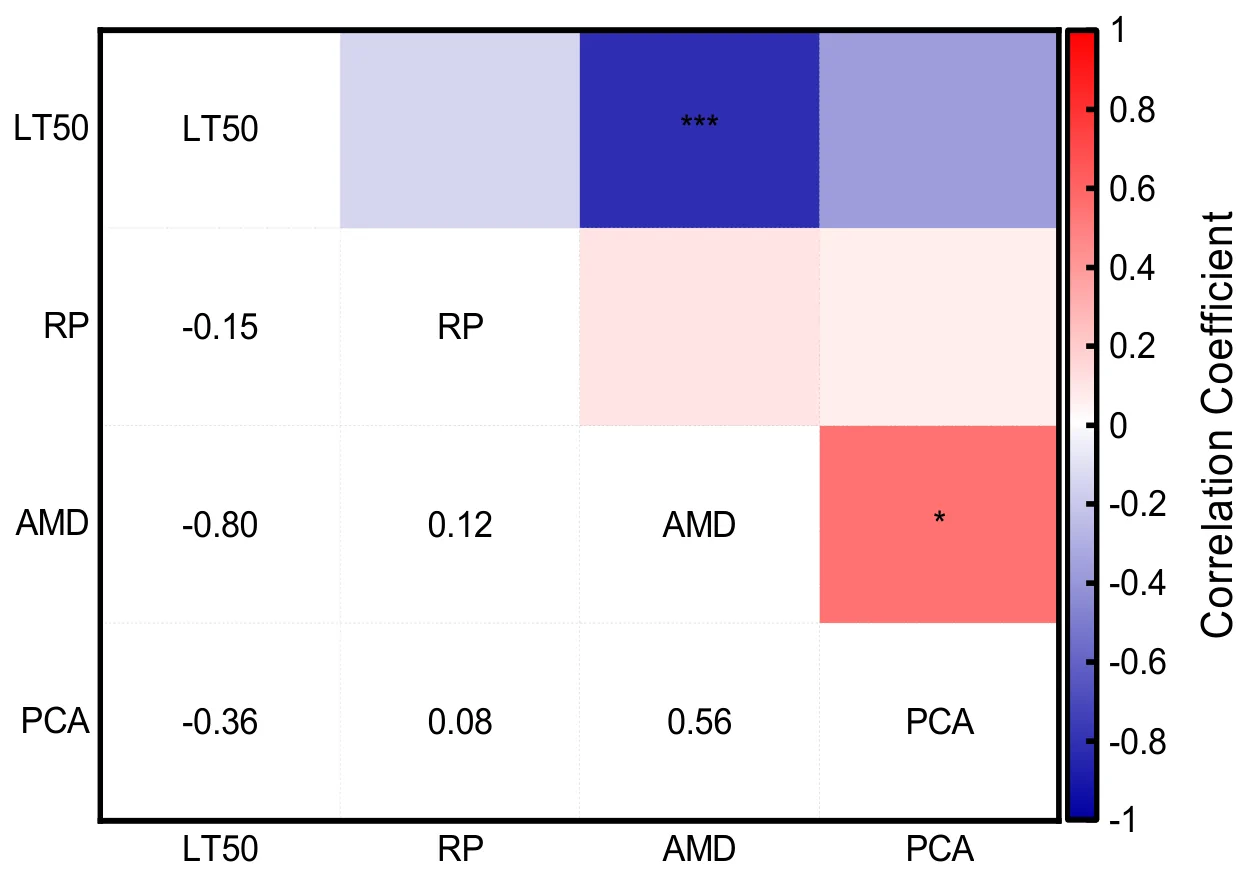
Pearson correlation matrix among the four core cold-tolerance evaluation indices in sweet cherry annual shoots. LT_50_: half-lethal temperature; RP: relative recovery growth percentage; AMD: average membership degree; PCA: principal component analysis score. The scale bar on the right represents the Pearson correlation coefficient (*r*), where red indicates a positive correlation and blue indicates a negative correlation. Asterisks indicate statistical significance: * *p* < 0.05, *** *p* < 0.001.

To systematically elucidate the relationships among thermodynamic tolerance, physiological regulation, and poststress recovery capacity in dormant sweet cherry annual shoots under low-temperature stress, a Pearson correlation analysis was performed on the four core evaluation indices: half-lethal temperature (LT_50_*)*, relative recovery growth percentage (RP), average membership degree (AMD), and PCA-based evaluation score (PCA) (Figure 7). The results indicated a strong, highly significant negative correlation between LT_50_ and AMD (*r* = −0.80, *p* < 0.001). Given that a lower LT_50_ value indicates better cold hardiness, this negative correlation validates the consistency between the thermodynamic survival limits and the cumulative physiological barrier represented by the AMD. Furthermore, a significant positive correlation was observed between the AMD and PCA scores (*r* = 0.56, *p* < 0.05). This convergence suggests that despite the use of distinct mathematical algorithms, both methods effectively capture similar trends in the physiological response of sweet cherry cultivars to cold stress. Conversely, the RP showed weak and statistically nonsignificant correlations with the LT_50_ (*r* = −0.05), AMD (*r* = −0.09), and PCA scores (*r* = −0.14) (*p* > 0.05). This statistical decoupling suggests that passive survival under extreme cold (regulated by LT_50_, AMD, and PCA) and active regenerative growth post-freezing (regulated by RP) represent two biologically distinct and independent processes in dormant sweet cherry. This separation highlights the ecological physiological trade-offs adopted by different cultivars under stress: certain cultivars prioritize resource allocation toward enhancing physical survival limits (indicated by lower LT_50_), whereas others favor rapid bud sprouting and nutritional reconstruction following cold damage (indicated by higher RP). These findings fully justify the multimethod integration strategy used in this study, as no single-dimensional evaluation index can simultaneously encompass these two independent physiological dimensions. The composite assessment model successfully bridges this gap, establishing a scientific balance between passive cold defense and postdamage recovery.

## 3. Discussion

### 3.1. Membrane Integrity and Lipid Peroxidation Under Cold Stress

The cell membrane acts as the primary barrier regulating mass exchange and signal transduction between the plant cell and its environment [22]. Under low-temperature stress, the rigidification of membrane lipids triggers a transition from a liquid-crystalline state to a solid-gel state, compromising selective permeability and leading to intracellular electrolyte leakage [23]. In our study, the relative electrical conductivity (REC) of the 16 sweet cherry annual shoots exhibited a typical sigmoidal increase with declining temperatures (Section 2.2.1). This outcome is consistent with that of Zhou et al. [24], who reported a gradual increase in electrolyte leakage in European plum twigs during progressive freezing, indicating that the initial stage of cold stress causes minor, reversible membrane damage before the threshold of severe physical rupture is crossed.

Furthermore, cold injury is tightly linked to lipid peroxidation driven by reactive oxygen species (ROS) [17]. As a cytotoxic end-product of lipid peroxidation, the accumulation of malondialdehyde (MDA) reflects the level of oxidative degradation of cell membranes [25]. Pradhan et al. [26] and Qin et al. [27] reported that compared with sensitive genotypes, cold-tolerant genotypes (e.g., in papaya and nectarine) consistently maintain lower MDA levels and membrane permeability, indicating that genetic variations in membrane cryostability dictate survival. Our results demonstrate that as temperatures decreased below −20 °C, the REC and MDA of cold-sensitive sweet cherry cultivars such as Kordia and Summit sharply and irreversibly increased, indicating a catastrophic loss of membrane integrity. Conversely, Tier I cultivars (Russia 8 and Reid) maintained flat MDA fluctuations and low REC increments, confirming their robust membrane protection and superior cryostability.

### 3.2. Osmoprotectant Accumulation and Osmosensing Efficiency

Osmoregulation is a critical biochemical adaptation that prevents freeze-induced cell dehydration [28]. Soluble sugars, soluble proteins, and proline act as compatible solutes that depress the cellular freezing point, stabilize the tertiary structure of macromolecules, and maintain cellular turgor [29]. In this study, the concentrations of all three osmolytes in sweet cherry twigs increased significantly with decreasing temperature (Section 2.3). This progressive accumulation aligns with the findings of Zhu et al. [30] on plum-apricot hybrids and Yooyongwech et al. [31] on peach buds, suggesting a conserved osmotic defense mechanism among Prunus species.

However, distinct intercultivar differences in the velocity and magnitude of osmolyte accumulation were observed. Cold-tolerant cultivars (Russia 8 and Reid) exhibited a highly sensitive sensing response, initiating rapid accumulation at mild freezing temperatures and maintaining elevated levels even under extreme cold. In contrast, sensitive cultivars (Kordia and Summit) displayed limited biosynthetic capacity and slower response times, leaving their tissues vulnerable to dehydration. These findings indicate that the cooperative accumulation and mobilization efficiency of osmoregulatory substances serve as important physiological indicators for screening cold-resistant sweet cherry germplasms.

### 3.3. Antioxidant Enzyme Dynamics and the “Single-Peak” Acclimation Response

Under low temperatures, plants rely on their antioxidant enzyme system to scavenge excess ROS and maintain cellular redox homeostasis [21]. Superoxide dismutase (SOD) initiates this response by converting superoxide radicals (O_2_^·−^) into hydrogen peroxide (H_2_O_2_), which is subsequently reduced to water and oxygen by peroxidase (POD) and catalase (CAT) [32]. In our study, the SOD, POD, and CAT activities in sweet cherry twigs followed a distinct “single-peak” (bell-shaped) curve with decreasing temperature (Section 2.4). This characteristic pattern reflects a transition from stress-induced physiological compensation to severe freezing damage.

At temperatures between −15 °C and −18 °C, the activity of antioxidant enzymes strongly increased to scavenge cold-induced ROS [33]. However, once temperatures breached the critical low-temperature tolerance threshold (specifically below −20 °C), these enzymatic activities plummeted. This post-peak decline is primarily attributable to low-temperature-induced macromolecular collapse, including structural protein denaturation, ribosomal assembly arrest, or generalized metabolic exhaustion within the damaged cells [34]. Similar “single-peak” enzymatic responses have been reported in apple twigs [35] and citrus [36] under freezing stress. Notably, compared with sensitive cultivars, cold-tolerant cherry cultivars exhibited earlier induction, higher peak activities, and sustained enzymatic stability at ultralow temperatures. These findings suggest that the thermal stability of antioxidant enzymes and their induction sensitivity at critical temperatures are key factors governing cold tolerance in sweet cherry.

### 3.4. Physiological Decoupling of Passive Survival and Poststress Recovery Growth

A major physiological revelation in this study is the statistical decoupling (lack of significant correlation) between the relative recovery growth percentage (RP) and immediate winter cell survival indices (LT_50_, MDA, and antioxidant enzymes) (Section 2.9). Under cold stress, woody plants typically adopt elaborate metabolic trade-offs governing resource allocation between growth processes and environmental defense adaptation [37].

The classical survival threshold (LT_50_) and membrane damage indices (REC, MDA) reflect the physical stress tolerance limit of parenchymal cells during static winter dormancy. However, poststress recovery (RP) is a dynamic process reflecting the regenerative capacity of cambial cells and dormant buds when permissive growth conditions return in spring [38]. The rapid regrowth of shoots relies on the reactivation of the vascular cambium, cell division, auxin translocation, and the mobilization of starch reserves stored in the xylem ray parenchyma to support new bud bursts [39]. Varieties such as ‘Luyu’ showed moderate winter membrane survival (*N*(LT_50_) = 0.4578) but exhibited the maximum recovery growth percentage (*N*(RP) = 1.0000). These findings suggest that despite experiencing partial cellular damage during freezing, Luyu possesses highly active dormant vegetative buds and a highly efficient vascular tissue repair system. Conversely, some cultivars with high static winter survival may undergo slow bud break because of sluggish hormone mobilization or depletion of nonstructural carbohydrate reserves [40]. This phenotypic divergence highlights that static winter survival and spring regenerative vitality are controlled by distinct physiological pathways, indicating that poststress recovery is an independent and indispensable dimension of plant cold hardiness evaluation.

### 3.5. Methodological Innovation of CRITIC Weighting and Agronomic Applications

Evaluating plant stress tolerance on the basis of a single parameter often leads to bias because of biological complexity. Although multi-index membership function analysis (MFA) and principal component analysis (PCA) are widely used to integrate multidimensional data, traditional MFA typically applies equal weights to all indices [41]. In plant biology, stress-induced metabolic pathways are highly interconnected; for instance, REC and MDA both reflect membrane damage, whereas SOD, CAT, and POD are co-linear parameters of the cellular antioxidant system. Applying equal weights to these co-linear parameters introduces redundant, hidden duplication that skews the final cumulative evaluation [42].

To address this issue, we utilized the CRITIC method, which assigns weights objectively on the basis of the contrast intensity (standard deviation) and conflict (correlation) of each index [18]. Under this model, because LT_50_ and membership degree (MD) represent overlapping physiological responses to cell injury, they were allocated moderate weights (21.66% and 21.94%, respectively). In contrast, RP-which represents the independent physiological dimension of poststress regrowth-was assigned the highest objective weight (32.11%) because of its low correlation (high conflict) with the other variables.

Using this model, the 16 cultivars were classified into four distinct tiers:

Tier I (Russia 8, Reid, Pacific Red, Brooks): Recommended for high-latitude cold regions because of their balanced active/passive defenses. Notably, Russia 8 is well documented for its superior winter hardiness in northern climates [43].

Tier II (e.g., Luyu): Genotypes with high recovery capacity (RP). The CRITIC method prevented Luyu from being misclassified into a lower tier, highlighting its suitability for regions prone to early spring late frost.

Tier III (Summit and Kordia): Susceptible to both membrane damage and slow recovery. The cultivation of these varieties requires agrotechnical interventions (e.g., wind machines and chemical cryoprotectants) to prevent winter injury.

Finally, it should be noted that this study primarily focused on evaluating the winter cold tolerance of dormant one-year-old shoots, as persistent winter freezing is the primary limiting factor for sweet cherry cultivation and regional distribution in Jiashi, Xinjiang, China. Consequently, the physiological and biochemical responses of flower buds, full-bloom floral organs, and young fruits under low-temperature stress were not determined, which represents a limitation of the current study. Since recurrent spring frosts during flowering also affect production, future research will simulate low-temperature stress on these reproductive organs to comprehensively analyze the cold hardiness traits across the entire phenological and growth cycle of sweet cherries.

## 4. Materials and Methods

### 4.1. Plant Materials and Experimental Site

The 16 sweet cherry cultivars used in this study were sourced from the cherry germplasm collection located in Toshkanla Village, Xiaputule Town, Jiashi County, Kashi Prefecture, Xinjiang, China (39°46′ N, 76°61′ E). The site is situated at an elevation of 1208.6 m and features a warm-temperate continental arid climate. The meteorological records indicate an annual mean temperature of 11.7 °C, with mean January and July temperatures of −5.4 °C and 25.6 °C, respectively, and an annual accumulated temperature ≥ 10 °C of 4400 °C.

For each cultivar, 200 healthy, uniform trees with comparable vigor and no visible signs of disease or pest infestation were selected as experimental subjects. Field management practices—including soil type, irrigation-fertilization (implemented via integrated water–fertilizer application), and routine pest and disease control—were standardized across all the cultivars to minimize confounding environmental and agronomic variability in the physiological measurements. Detailed information on the 16 cultivars is provided in Table 8.

### 4.2. Experimental Design and Low-Temperature Treatments

On 10 December 2025, a total of 30 healthy one-year-old dormant branches (15~20 cm in length, 0.8~1.3 cm in diameter, of uniform thickness) were collected from the peripheral middle canopy of the trees for each sweet cherry cultivar. The gathered branches were rinsed with distilled water, and both cut ends were immediately sealed with paraffin wax to prevent industrial dehydration. Branch samples were then transported to the laboratory and stored at 5 °C prior to treatment. For each biochemical index (MDA, osmotic substances, enzyme activities), 3 biological replicates were analyzed per cultivar per temperature treatment, with each replicate consisting of tissues pooled from 5 independent shoots.

The experiment involved nine temperature gradient treatments: 5 °C (control), 0 °C, −5 °C, −10 °C, −15 °C, −18 °C, −20 °C, −22 °C, and −25 °C. The temperature in the temperature-controlled incubator was decreased at a constant cooling rate of 4 °C/h. Upon reaching each target temperature, the samples were kept at that temperature for 12 h in a benchtop high and low temperature test chamber (SW4005, Shanghai Jianheng Instrument Co., Ltd., Shanghai, China). Afterward, the branches were gradually rewarmed to 5 °C at the same rate before the subsequent physiological and biochemical evaluations.

### 4.3. Parameters and Methods

#### 4.3.1. Relative Conductivity and Lethal Temperature

The relative electrical conductivity (REC) of the shoots was determined using an electrical conductivity meter (FiveEasy Plus FE28 conductivity meter, Mettler-Toledo Instruments (Shanghai) Co., Ltd., Shanghai, China). Briefly, the shoots subjected to low-temperature treatments were cut into segments of approximately 0.5 cm. A sample of approximately 1.0 g was weighed and placed into a 50 mL test tube containing 25 mL of deionized water. The tubes were shaken on constant temperature shaker (Model ZWYR-240, Shanghai Zhicheng Analytical Instrument Manufacturing Co., Ltd., Shanghai, China) at 25 °C for 90 min, after which the initial electrical conductivity (*EC*_1_) was recorded. The tubes were subsequently heated in a boiling water bath (Model HH-S8, Jiangsu Jinyi Instrument Technology Co., Ltd., Jiangsu, China) for 20 min to achieve complete electrolyte release. After the samples cooled to room temperature, the final electrical conductivity (*EC*_2_) was measured. The REC was calculated using the following equation:

(3) $$REC(\%)=\frac{{EC}_{1}}{{EC}_{2}}\times 100$$

Relative electrical conductivity (REC) data at various temperatures were used to determine the semilethal temperature (LT_50_). The relationship between the treatment temperature (*x*) and the REC(*y*) was fitted to a sigmoidal logistic regression model:

(4) $$y=A+\frac{B}{1+e^{(x+C)/D}}$$

where *y* represents the relative electrical conductivity (%); *x* represents the treatment temperature (°C); *A* is the lower asymptote; *B* is the range of REC increase; *C* is the parameter defining the inflection point; and *D* is the slope factor. The LT_50_ was determined as the temperature of the inflection point and was calculated as −*C* (°C).

#### 4.3.2. Determination of Physiological Indices

The contents of soluble sugar (SS), soluble protein (SP), proline (Pro), and malondialdehyde (MDA), as well as the activities of superoxide dismutase (SOD), peroxidase (POD), and catalase (CAT), were determined using specific commercial assay kits purchased from Shanghai Enzyme-linked Biotechnology Co., Ltd. (Shanghai, China) according to the manufacturer’s instructions. The specific kits used were as follows: Soluble Sugar Assay Kit (Cat# ML-W-Y-108), Soluble Protein Assay Kit (BCA Method, Cat# ML-W-Y-201), Proline Assay Kit (Cat# ML-W-Y-305), Malondialdehyde (MDA) Assay Kit (Cat# ML-W-Y-102), Superoxide Dismutase (SOD) Activity Assay Kit (Cat# ML-W-Y-402), Peroxidase (POD) Activity Assay Kit (Cat# ML-W-Y-405), and Catalase (CAT) Activity Assay Kit (Cat# ML-W-Y-409). All spectrophotometric measurements during determination were performed using a multispectral microplate reader (Model HBS-ScanA, Nanjing Detie Experimental Equipment Co., Ltd., Nanjing, China).

#### 4.3.3. Recovery Growth Percentage

To evaluate their growth percentage (RP), shoots subjected to different low-temperature treatments were cut diagonally at their lower ends and inserted into Erlenmeyer flasks. The flasks were then placed in an intelligent illumination incubator (Model MGC-350HP, Shanghai Yiheng Scientific Instrument Co., Ltd., Shanghai, China) and cultivated at 25 °C, and the water was replaced every 2 d. The percentage of viable buds on day 28 was calculated as an indicator of the ability of this cherry variety to resume growth under low-temperature stress conditions. The RGR was calculated using the following equation:

(5) $$RGR(\%)=\frac{N_{s}}{N_{t}}\times 100$$

where *N_s_* represents the number of sprouted buds and *N_t_* represents the total number of buds.

### 4.4. Data Analysis and Comprehensive Evaluation

The cold tolerance of the 16 sweet cherry cultivars was comprehensively evaluated using the membership function analysis method. The relative electrical conductivity (REC) at various temperatures was fitted to a logistic regression model to determine the semilethal temperature (LT_50_). Data processing, error analysis, and significance testing were performed using SPSS software (version 26.0, IBM Corp., Armonk, NY, USA).

The membership function values were calculated as follows:

To eliminate the differences in dimensions and magnitudes among different evaluation indices, the raw experimental data were first standardized using min-max normalization:

(6) $${X^{\prime }}_{ij}=\frac{X_{ij}-X_{jmin}}{X_{jmax}-X_{jmin}}$$

where *X_ij_* represents the measured value of the j-th index of the i-th cultivar; *X_jmax_* and *X_jmin_* represent the maximum and minimum values of the j-th index across all cultivars, respectively; and *X^′^_ij_* is the standardized value ranging from 0 to 1.

Subsequently, the membership function values *U*(*X_ij_*) were calculated. For indicators positively correlated with cold tolerance (e.g., SOD, POD, and CAT activities, and proline, soluble sugar, and soluble protein contents), the membership function value was defined as:

(7) $$U(X_{ij})=X_{ij}^{\prime }$$

For indicators negatively correlated with cold tolerance (e.g., MDA content and REC), the value was calculated as:

(8) $$U(X_{ij})=1-X_{ij}^{\prime }$$

where *U*(*X_ij_*) represents the membership function value of the j-th index of the i-th cultivar.

For the LT_50_ index, a positive transformation was performed prior to normalization. To avoid potential hidden weighting caused by overlapping information among physiological and biochemical indices, the criteria importance through intercriteria correlation (CRITIC) method was used for objective weight allocation. The information content (*C_j_*) of each normalized indicator was calculated on the basis of its contrast intensity (represented by standard deviation, *S_j_*) and conflict (represented by correlation coefficients with other indicators, *r_ij_*):

(9) $$C_{j}=S_{j}\sum_{i=1}^{n}(1-r_{ij})$$

where *S_j_* is the standard deviation of the *j*-th indicator and *r_ij_* represents the Pearson correlation coefficient between the *i*-th and *j*-th indicators.

The objective weight (*W_j_*) of the *j*-th indicator is then calculated as follows:

(10) $$W_{j}=\frac{C_{j}}{\sum_{j=1}^{m}C_{j}}$$

Finally, the comprehensive cold tolerance score (*F* score) for each cultivar was calculated using the weighted sum of the standardized indicators:

(11) $$Comprehensive\;Score(F)=\sum_{j=1}^{m}\left(W_{j}\times X_{std\_j}\right)$$

where *X_std_j_* represents the standardized value of the *j*-th indicator (i.e., *N*(LT_50_), *N*(MD), *N*(RP), and *N*(PCA)).

## 5. Conclusions

In this study, the physiological mechanisms and cold tolerance of 16 sweet cherry cultivars were comprehensively evaluated using annual dormant shoots subjected to a simulated low-temperature gradient (5 °C to −25 °C). A multidimensional evaluation framework integrating the semilethal temperature (LT_50_), average membership degree (AMD), relative recovery growth Percentage (RP), and PCA-based scores was constructed using the objective CRITIC weighting method. This approach assigned the highest weight (32.11%) to RP because of its statistical independence from static survival indicators, thereby resolving a critical limitation of conventional single-index assessments.

The key conclusions are as follows:

(1) Cold-tolerant cultivars (e.g., Russia 8, Reid, Pacific Red, and Brooks) exhibited superior membrane integrity (lowest REC and MDA at −25 °C), robust osmotic adjustment (high proline, soluble sugar, and protein accumulation), and sustained antioxidant enzyme activity (SOD, POD, and CAT) even under extreme cold stress.
(2) Crucially, RP was not significantly correlated with the LT_50_, AMD, or PCA score (*p* > 0.05), demonstrating that passive survival under freezing stress and active poststress regenerative capacity are two biologically distinct and independent physiological processes in dormant sweet cherry—highlighting an essential trade-off in resource allocation between winter survival and spring recovery.
(3) Based on the CRITIC-weighted comprehensive *F*-score, the 16 cultivars were unambiguously classified into four tiers:

Tier I (Highly Cold-Tolerant; *F* ≥ 0.65): Russia 8 (0.8629), Reid (0.7334), Pacific Red (0.7056), and Brooks (0.6943)—ideal for high-latitude regions with severe winter freezing due to balanced defense mechanisms;

Tier II (Moderately to Highly Cold-Tolerant; 0.50 ≤ *F* < 0.65): Tieton, Sandra Rose, Luyu, Rivedel—notably, Luyu (*F* = 0.5393, *N*(*RP*) = 1.0000) ranks highly because of exceptional recovery capacity, making it a top candidate for regions prone to spring late frost;

Tier III (Moderately to Weakly Cold-Tolerant; 0.30 ≤ *F* < 0.50): Jiahong, Rocket, Tamara, Lapins, Frisco, Taisho-nishiki;

Tier IV (Cold-Sensitive; *F* < 0.30): Summit (0.2451) and Kordia (0.2024), with the latter being the most vulnerable because of poor membrane cryoprotection (*N*(LT_50_) = 0.0000) and a delayed osmoregulatory response (*N*(*MD*) = 0.0644).

This four-dimensional model provides a scientifically robust, agronomically actionable foundation for cultivar selection in cold-temperate regions and highlights that future breeding programs must explicitly target both winter survival and spring recovery traits to increase climate resilience in sweet cherry production.

## Figures and Tables

**Table 1:** Logistic regression equations, determination coefficients (*R^2^*), and semilethal temperatures (LT_50_) of annual shoots from 16 sweet cherry cultivars under low-temperature stress.

Variety Name	Fitting Equation	Fitting Value	LT_50_ ± SE/°C	Sort
Summit	$y=35.12+\frac{19.74}{1+e^{\left(x+18.53/2.47\right)}}$	0.993	−18.53 ± 0.22	15
Lapins	$y=38.15+\frac{13.81}{1+e^{\left(x+21.82/3.01\right)}}$	0.976	−21.82 ± 0.43	9
Tamara	$y=35.23+\frac{19.86}{1+e^{\left(x+21.43/2.85\right)}}$	0.985	−21.43 ± 0.35	10
Russia 8	$y=38.21+\frac{13.13}{1+e^{\left(x+25.02/2.14\right)}}$	0.941	−25.02 ± 0.51	2
Rivedel	$y=41.27+\frac{14.85}{1+e^{\left(x+19.58/2.36\right)}}$	0.968	−19.58 ± 0.41	14
Luyu	$y=36.58+\frac{26.63}{1+e^{\left(x+21.06/3.52\right)}}$	0.995	−21.06 ± 0.18	12
Jiahong	$y=34.06+\frac{19.76}{1+e^{\left(x+21.89/3.22\right)}}$	0.991	−21.89 ± 0.24	8
Rocket	$y=36.87+\frac{16.07}{1+e^{\left(x+22.83/2.95\right)}}$	0.959	−22.83 ± 0.48	6
Reid	$y=35.43+\frac{20.64}{1+e^{\left(x+23.68/2.78\right)}}$	0.982	−23.68 ± 0.38	4
Frisco	$y=33.41+\frac{24.42}{1+e^{\left(x+23.14/3.31\right)}}$	0.977	−23.14 ± 0.42	5
Taisho-nishiki	$y=39.30+\frac{12.86}{1+e^{\left(x+21.34/2.93\right)}}$	0.982	−21.34 ± 0.39	11
Brooks	$y=33.87+\frac{18.19}{1+e^{\left(x+24.48/2.21\right)}}$	0.963	−24.48 ± 0.44	3
Pacific Red	$y=39.04+\frac{9.92}{1+e^{\left(x+25.74/2.06\right)}}$	0.954	−25.74 ± 0.61	1
Tieton	$y=37.08+\frac{18.26}{1+e^{\left(x+21.01/3.45\right)}}$	0.994	−21.01 ± 0.19	13
Sandra Rose	$y=36.21+\frac{18.46}{1+e^{\left(x+22.27/3.11\right)}}$	0.989	−22.27 ± 0.28	7
Kordia	$y=43.27+\frac{13.15}{1+e^{\left(x+17.08/2.12\right)}}$	0.990	−17.08 ± 0.26	16

**Table 2:** Membership function values of the physiological indices and comprehensive cold tolerance rankings of 16 sweet cherry cultivars.

Variety Name	Derivative								Average Membership	Sort
	REC	MDA	SP	SS	Pro	SOD	POD	CAT		
Summit	0.074	0.000	0.277	0.212	0.628	0.406	0.340	0.000	0.242	16
Lapins	0.453	0.684	0.000	0.294	0.183	0.248	0.526	0.080	0.309	14
Tamara	0.477	0.316	0.155	0.430	0.229	0.594	0.371	0.272	0.355	12
Russia 8	0.455	0.316	0.751	0.839	1.000	0.805	0.412	0.580	0.645	2
Rivedel	0.621	0.264	0.385	0.330	0.511	0.591	0.567	0.253	0.440	8
Luyu	0.411	0.264	0.830	0.725	0.129	0.138	0.423	0.451	0.421	11
Jiahong	0.500	0.528	0.077	0.665	0.015	0.597	1.000	0.407	0.474	7
Rocket	0.308	0.316	0.387	0.546	0.118	0.359	0.371	0.340	0.343	13
Reid	0.675	0.368	0.943	0.999	0.187	0.000	0.753	0.401	0.541	4
Frisco	1.000	0.104	0.167	0.178	0.486	0.735	0.155	0.574	0.425	10
Taisho-nishiki	0.599	0.156	0.620	0.219	0.559	0.557	0.619	0.580	0.489	6
Brooks	0.772	0.472	0.551	0.689	0.632	1.000	0.000	0.377	0.562	3
Pacific Red	0.526	0.580	0.740	0.590	0.688	0.909	0.258	0.999	0.661	1
Tieton	0.822	0.368	0.498	0.711	0.052	0.477	0.176	0.839	0.493	5
Sandra Rose	0.219	1.000	0.866	0.706	0.123	0.198	0.072	0.222	0.426	9
Kordia	0.000	0.368	0.376	0.001	0.000	0.661	0.155	0.592	0.269	15

**Table 3:** Effects of Low-temperature Stress on the Relative Recovery Growth percentage of Dormant Annual Shoots.

Variety	Recovery Growth Percentage/%									−15 °C~−25 °C Average	Sort
	5 °C	0 °C	−5 °C	−10 °C	−15 °C	−18 °C	−20 °C	−22 °C	−25 °C		
Summit	100.0	100.0	100.0	88.0	78.3	66.3	57.8	26.5	0.0	45.78	10
Lapins	100.0	97.6	97.6	84.7	84.7	71.8	51.8	18.8	0.0	45.42	11
Tamara	100.0	87.1	87.1	81.7	75.3	64.5	49.5	26.9	0.0	43.24	13
Russia 8	100.0	75.0	75.0	75.0	67.7	59.4	52.1	33.3	18.8	46.26	9
Rivedel	100.0	98.9	98.9	94.3	83.0	75.0	55.7	30.7	0.0	48.88	5
Luyu	100.0	88.3	83.1	83.1	75.0	64.9	63.6	49.4	25.0	55.58	1
Jiahong	100.0	87.2	84.9	79.1	76.7	60.5	54.7	26.7	0.0	43.72	12
Rocket	100.0	91.6	91.6	91.6	77.1	69.9	60.2	39.8	12.0	51.8	3
Reid	100.0	95.0	87.0	80.0	77.0	72.0	52.0	38.0	14.0	50.6	4
Frisco	100.0	93.6	81.9	80.9	56.4	52.1	45.7	28.7	0.0	36.58	15
Taisho-nishiki	100.0	100.0	92.5	82.5	52.9	37.5	33.8	13.8	0.0	29.28	16
Brooks	100.0	92.0	84.0	77.0	74.0	63.0	50.0	34.0	20.0	48.2	6
Pacific Red	100.0	88.0	74.0	74.0	70.0	61.0	54.0	35.0	20.0	48	7
Tieton	100.0	97.6	97.6	87.8	84.1	70.7	53.7	43.9	15.9	53.66	2
Sandra Rose	100.0	89.8	83.0	73.9	66.7	62.5	53.4	37.5	18.8	47.78	8
Kordia	100.0	92.8	84.3	79.5	68.7	57.8	44.6	33.7	0.0	40.96	14

**Table 4:** Principal component eigenvectors and contribution rates.

Index	PC1	PC2	PC3	PC4
Relative electrical conductivity	−0.243	0.349	−0.306	0.655
Soluble protein	0.191	0.110	0.777	0.401
Soluble sugar	0.336	0.533	−0.006	−0.257
Proline	0.473	0.083	0.163	−0.253
Malonaldehyde	−0.373	0.358	0.260	−0.350
CAT	0.417	−0.193	0.065	0.374
POD	−0.430	0.229	0.370	0.065
SOD	−0.265	−0.597	0.258	−0.114
Eigenvalue	2.852	1.503	1.061	0.941
Contribution rate (%)	35.649	18.789	13.262	11.765
Cumulative contribution rate (%)	35.649	54.439	67.700	79.466

**Table 5:** Overall scores and cold hardiness rankings of 16 sweet cherry varieties.

Variety	*F*1	*F*2	*F*3	*F*4	*F*	Sort
Summit	−1.251	−0.052	−2.211	−0.192	−0.971	15
Lapins	−0.393	0.640	−0.843	−0.390	−0.223	9
Tamara	0.536	−1.133	−0.046	1.903	0.247	6
Russia 8	4.062	1.849	0.566	−1.002	2.205	1
Rivedel	0.306	2.305	−0.817	0.137	0.566	4
Luyu	−2.907	1.928	0.794	−0.037	−0.721	14
Jiahong	−1.290	0.321	1.388	0.169	−0.246	10
Rocket	−1.261	−0.820	−0.829	−1.099	−1.061	16
Reid	1.387	0.703	0.071	0.749	0.911	2
Frisco	−0.540	−1.326	0.214	−1.014	−0.670	13
Taisho-nishiki	−0.680	−0.429	1.233	0.531	−0.122	8
Brooks	1.384	−1.267	1.076	−0.431	0.437	5
Pacific Red	−0.676	−1.109	0.595	−1.136	−0.635	12
Tieton	0.433	−0.857	−0.332	1.295	0.128	7
Sandra Rose	2.290	−0.796	−0.902	−0.055	0.680	3
Kordia	−1.401	0.044	0.044	0.571	−0.526	11

**Table 6:** Comprehensive evaluation of the cold tolerance of 16 sweet cherry cultivars.

Variety	*N*(LT_50_)	*N*(MD)	*N*(RP)	*N*(PCA)	*F*-Score	Sort
Summit	0.1325	0.0000	0.6532	0.0276	0.2451	15
Lapins	0.5301	0.1599	0.6389	0.2566	0.4174	12
Tamara	0.4819	0.2697	0.5527	0.4005	0.4383	11
Russia 8	0.8916	0.9618	0.6722	1.0000	0.8629	1
Rivedel	0.2530	0.4726	0.7760	0.4982	0.5287	8
Luyu	0.4578	0.4272	1.0000	0.1041	0.5393	7
Jiahong	0.5422	0.5537	0.5717	0.2495	0.4831	9
Rocket	0.6265	0.2411	0.8915	0.0000	0.4749	10
Reid	0.7349	0.7136	0.8440	0.6038	0.7334	2
Frisco	0.6627	0.4368	0.2890	0.1197	0.3613	13
Taisho-nishiki	0.4699	0.5895	0.0000	0.2875	0.3010	14
Brooks	0.8072	0.7637	0.7490	0.4587	0.6943	4
Pacific Red	1.0000	1.0000	0.7411	0.1304	0.7056	3
Tieton	0.4337	0.5990	0.9652	0.3641	0.6237	5
Sandra Rose	0.5904	0.4391	0.6762	0.5331	0.5708	6
Kordia	0.0000	0.0644	0.4624	0.1638	0.2024	16

**Table 7:** Classification of the 16 sweet cherry cultivars into cold-tolerance tiers based on comprehensive evaluation F-scores.

Tier	Classfication	Cultivars	F-Score Range	Overal Ranking
Tier I	Highly Cold-Tolerant	Russia 8	F ≥ 0.65	1.
		Reid		2.
		Pacific Red		3.
		Brooks		4.
Tier II	Moderately to Highly Cold-Tolerant	Tieton	0.50 ≤ *F* < 0.65	5.
		Sandra Rose		6.
		Luyu		7.
		Rivedel		8.
Tier III	Moderately to Weakly Cold-Tolerant	Jiahong	0.30 ≤ *F* < 0.50	9.
		Rocket		10.
		Tamara		11.
		Lapins		12.
		Frisco		13.
		Taisho-nishiki		14.
Tier IV	Cold-Sensitive	Summit	*F* < 0.30	15.
		Kordia		16.

**Table 8:** List of materials.

Variety Code	Variety Name	Origin	Tree Vigor	Precocity	Self-Fertility	Fruit Weight and Quality
1	Summit	Canada	Strong vigor, upright-spreading	Moderate	Self-incompatible	Very large (10–12 g); Heart shaped, dark red, firm, sweet flavor.
2	Lapins	Canada	Strong vigor, upright	Highly precocious	Self-fertile	Large (9–11 g); Round heart shaped, mahogany, firm, sweet.
3	Tamara	Czech Republic	Moderate-strong vigor, spreading	Precocious	Self-incompatible	Exceptionally large (12–14 g); Dark red, very firm, juicy, sweet and subacid.
4	Russia 8	Russia	Strong vigor, spreading	Precocious	Self-incompatible	Very large (10–12 g); Dark red, firm, thick skin, highly sweet flavor.
5	Rivedel	France	Moderate vigor, upright-spreading	Precocious	Self-incompatible	Medium large (7–9 g); Heart shaped, dark red, soft medium flesh, sweet.
6	Luyu	Shandong, China	Strong vigor, upright-spreading	Precocious	Self-incompatible	Large (8–10 g); Broad heart shaped, bright red to dark red, soft medium, sweet.
7	Jiahong	Dalian, China	Strong vigor, spreading	Moderate	Self-incompatible	Large (8–10 g); Yellow orange with red blush, soft medium flesh, very sweet.
8	Rocket	United States	Strong vigor, upright-spreading	Highly precocious	Self-incompatible	Very large (10–12 g); Round heart shaped, dark red, very firm, early ripener.
9	Reid	Canada	Moderate vigor, spreading	Precocious	Self-fertile	Large (9–11 g); Dark red, firm, late season ripening, sweet.
10	Frisco	United States	Moderate to strong, upright-spreading	Precocious	Self-fertile/partially	Large (9–11 g); Kidney shaped, dark mahogany, firm, sweet, low acid.
11	Taisho-nishiki	Japan	Moderate-strong vigor, spreading	Moderate	Self-incompatible	Medium large (8–9 g); Bright red, juicy, soft firm, sweet and slightly sour.
12	Brooks	United States	Moderate vigor, spreading	Precocious	Self-incompatible	Large (9–10 g); Flat round, dark red, very firm, sweet tart, premium flavor.
13	Pacific Red	United States	Moderate vigor, upright-spreading	Highly precocious	Self-fertile	Large (9–11 g); Dark red, round heart shaped, very firm, sweet flavor.
14	Tieton	United States	Strong vigor, spreading	Precocious	Self-incompatible	Exceptionally large (11–13 g); Kidney shaped, dark red, very firm, sweet.
15	Sandra Rose	Canada	Moderate vigor, spreading	Precocious	Self-fertile	Very large (10–12 g); Round, dark red, moderately firm, exceptionally sweet.
16	Kordia	Czech Republic	Strong vigor, spreading	Moderate	Self-incompatible	Very large (10–12 g); Heart shaped, dark mahogany or black, very firm, sweet.

## Data Availability

The datasets generated and analysed during this study are maintained by the first author. The raw data can be obtained from the first author upon reasonable request.
